# Beyond Yield: Molecular Crop Quality as a Missing Dimension of Green Chemistry in Sustainable Agriculture

**DOI:** 10.3390/molecules31183261

**Published:** 2026-09-15

**Authors:** Maria Czernicka

**Affiliations:** Department of Bioenergetics, Food Analysis and Microbiology, Faculty of Technology and Life Sciences, University of Rzeszów, Zelwerowicza 8b St., 35-601 Rzeszow, Poland; mczernicka@ur.edu.pl

**Keywords:** green chemistry, molecular crop quality, nano-enabled biofortification, plant biostimulants, bioactive metabolites, secondary metabolism, green analytical chemistry

## Abstract

Green chemistry provides a conceptual and technological framework for redesigning agricultural inputs and analytical workflows toward safer, more efficient, and more sustainable crop production. In this review, molecular crop quality is proposed as a chemical and metabolomic endpoint of green chemistry in sustainable agriculture. Rather than evaluating agricultural interventions proposed as compatible with green chemistry only through yield, input reduction, or environmental performance, this review focuses on their capacity to reshape crop composition, including micronutrient density, primary metabolites, specialized metabolites, bioactive compounds, antioxidant potential, and bioaccessibility. Particular attention is given to nano-enabled biofortification, biogenic nanomaterials, plant-derived biostimulants, microbial consortia, and biodegradable carrier systems as tools that may modulate nutrient uptake, redox signaling, transcriptional regulation, and selected biosynthetic pathways. Representative changes in phenolics and flavonoids, carotenoids, glucosinolates, and capsaicinoids are discussed as examples of how agricultural interventions evaluated within a green chemistry framework can influence functional and nutritional traits at the molecular level. The review also considers green extraction and analytical platforms, including ultrasound-assisted extraction, microwave-assisted extraction, supercritical-fluid extraction, LC-MS, ICP-MS, NMR, and antioxidant assays, as essential tools for reliable assessment of molecular crop quality. Finally, safety-related challenges are critically examined, including nanoparticle characterization, dose-dependent phytotoxicity, environmental fate, bioaccessibility, and regulatory uncertainty. By integrating green chemistry, plant metabolism, nano-enabled biofortification, biostimulation, and analytical chemistry, this review outlines a framework for assessing crop quality beyond yield and highlights the need for standardized, mechanistically informed, and safety-oriented approaches to future agricultural innovation.

## 1. Introduction

Global food security is increasingly challenged by climate change, soil degradation, and micronutrient malnutrition affecting more than two billion people worldwide [1,2]. Hidden hunger, including deficiencies in essential micronutrients such as iron, zinc, and selenium, persists even in populations with adequate caloric intake, creating a need for biofortification strategies that are agronomically effective, nutritionally meaningful, and environmentally responsible [3,4]. At the same time, increasing attention is being directed toward plant-derived foods rich in phenolic compounds, carotenoids, glucosinolates, and other bioactive constituents [5,6]. Crop quality should therefore not be evaluated only through yield, external appearance, or basic nutrient composition. A broader perspective is needed in which molecular crop quality encompasses the mineral, primary-metabolite, specialized-metabolite, antioxidant-related, bioaccessibility, and safety characteristics of edible plant tissues. This molecular dimension directly connects agricultural input design with the nutritional, functional, and chemical quality of food.

Green chemistry provides a relevant framework for this transition by prioritizing waste prevention, resource and energy efficiency, renewable feedstocks, safer chemical design, and reduced toxicity [7,8,9]. Its application to agricultural systems may support the development of biogenic nanoparticles, plant-derived biostimulants, microbial formulations, biodegradable carriers, and analytical workflows with reduced solvent or energy demand [10,11,12,13]. However, descriptors such as green-synthesized, biogenic, natural, biodegradable, or nano-enabled refer only to selected properties of an intervention. They do not by themselves establish reduced environmental burden, improved resource efficiency, food safety, or superiority over an appropriate conventional alternative. Green chemistry compatibility in agriculture should therefore be treated as an evidence-based and multidimensional classification rather than as an intrinsic property of a material.

In this review, biogenic nanoparticles are defined as nanoscale materials synthesized wholly or partly through biological systems or biologically derived agents, such as plant extracts, microorganisms, or biomolecules acting as reducing, stabilizing, or capping agents. Nano-enabled biofortification refers to the use of nanoscale materials or nanoformulated nutrient-delivery systems to increase the concentration, availability, or nutritionally relevant chemical forms of essential elements in edible crop tissues. Nano-enabled nutrient delivery has received particular attention because particle-based formulations may modify nutrient dissolution, retention, uptake, and transport and may consequently influence both elemental accumulation and crop metabolism [14,15,16]. Biogenic synthesis using plant extracts, microorganisms, or agricultural residues can reduce the use of hazardous reducing or stabilizing agents, although the resulting materials still require physicochemical characterization and safety assessment [17,18]. Current evidence indicates that nano-enabled biofortification can increase Zn, Fe, Se, and other nutrients in selected edible crop tissues, but consistent superiority over ionic, chelated, or conventional formulations has been demonstrated only in specific crop-formulation systems [14,15,16].

Biostimulants and microbial inputs represent a related but mechanistically distinct intervention class. Under EU Regulation 2019/1009, plant biostimulants are substances or microorganisms that stimulate plant nutrition processes independently of their direct nutrient content [19]. Their effects may involve nutrient-use efficiency, root development, rhizosphere activity, stress tolerance, and crop-quality traits [20,21]. Combining these inputs with nanofertilizers or biodegradable delivery systems may produce complementary responses by coupling nutrient availability with broader physiological or microbial regulation [22,23]. Nevertheless, stronger responses from combined treatments should not be interpreted automatically as synergy, because this conclusion requires factorial designs that separate individual component effects and quantify their interaction.

Reliable interpretation of molecular crop quality also depends on the analytical workflow. Green extraction procedures, including ultrasound-assisted, microwave-assisted, and supercritical-fluid extraction, can reduce solvent use, processing time, or energy demand, but their suitability remains dependent on recovery, selectivity, compound stability, and the plant matrix examined [24,25]. Complementary analytical platforms such as LC-MS, ICP-MS, and NMR can provide compositional, elemental, and metabolomic information, yet differences in sampling, extraction, calibration, annotation, and endpoint selection may substantially affect study comparability [26,27]. Analytical sustainability should therefore not be considered separately from analytical reliability.

Despite increasing research activity, the evidence base remains fragmented. Major limitations include incomplete formulation characterization, insufficient dose-matched conventional comparators, uncertain attribution of effects in combined treatments, inconsistent analytical endpoints, limited assessment of speciation and bioaccessibility, and the predominance of short-term controlled-environment experiments [16,22,28,29]. Environmental fate, repeated exposure, non-target effects, food-chain transfer, and regulatory readiness also remain insufficiently resolved [30,31,32]. The central question is therefore not simply whether an intervention can alter crop composition, but whether it can produce a nutritionally relevant and reproducible molecular crop-quality benefit through a safer and more resource-efficient strategy without introducing disproportionate environmental or food-safety trade-offs.

**Figure 1:**
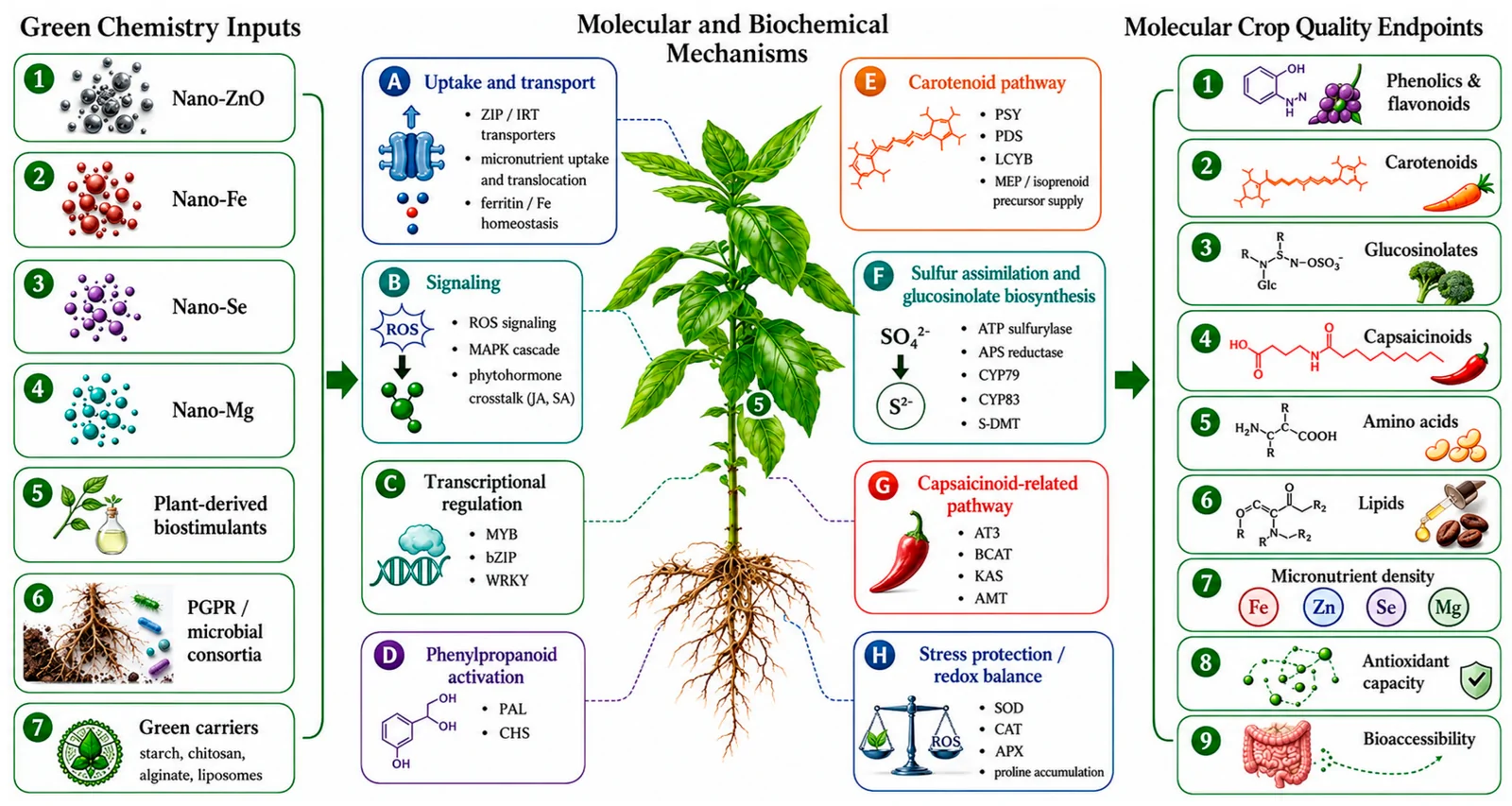
Conceptual framework linking agricultural inputs evaluated within a green chemistry framework with molecular crop-quality endpoints. Nano-enabled micronutrient delivery, plant-derived biostimulants, PGPR and microbial consortia, and biodegradable carrier systems may affect crop quality through nutrient uptake and transport, ROS/MAPK signaling, phytohormone crosstalk, transcriptional regulation, secondary-metabolite biosynthesis, and redox homeostasis. These processes are reflected in measurable quality endpoints, including micronutrient density, bioactive compound accumulation, antioxidant capacity, and bioaccessibility.

Accordingly, this structured critical narrative review aims to: (1) establish evidence-based criteria for evaluating the compatibility of agricultural interventions with green chemistry; (2) critically compare nano-enabled delivery, biostimulants, microbial inputs, biodegradable carriers, and combined formulations in relation to molecular crop quality; (3) examine the methodological and mechanistic limitations affecting evidence interpretation; and (4) integrate environmental fate, food-chain safety, comparative risk–benefit assessment, and translational priorities. Figure 1 summarizes this conceptual framework by linking candidate agricultural interventions with molecular and biochemical processes and measurable crop-quality endpoints. The mechanisms illustrated in the figure should be interpreted as plausible and context-dependent routes rather than as responses demonstrated universally for every intervention class. The novelty of this review lies in treating molecular crop quality as an explicit endpoint of green chemistry-compatible agricultural interventions and in evaluating these interventions through an integrated framework combining compositional benefit, comparative performance, environmental fate, food safety, and translational robustness.

## 2. Review Scope, Literature Identification, and Critical Appraisal

This article was designed as a critical narrative review rather than a systematic review or meta-analysis. It focused on agricultural interventions evaluated in relation to green chemistry principles and measurable effects on molecular crop quality, defined here as the mineral, biochemical, metabolomic, nutritional, and safety-related composition of edible plant tissues, including micronutrient density and speciation, primary and specialized metabolites, antioxidant-related traits, gastrointestinal bioaccessibility, and chemical safety.

Initial literature mapping was performed on 16 June 2026. The broad research question was iteratively expanded into topic-specific queries addressing green chemistry in agriculture, molecular crop quality, nano-enabled biofortification, biogenic nanomaterials, plant biostimulants, microbial inputs, biodegradable delivery systems, metabolomics, bioaccessibility, and environmental and food-safety aspects. This preliminary scoping identified 336 candidate publications, while backward and forward citation tracking identified a further 70 records, yielding an initial pool of 406 candidate publications. This pool was used to map the field, identify major thematic areas, and locate core evidence rather than as a formal systematic-review screening set.

The evidence base was subsequently refined through targeted searches in Scopus, Web of Science Core Collection, PubMed, and Google Scholar, together with continued backward and forward citation tracking. The final literature update was performed on 31 July 2026 and consisted of targeted updating of topic-specific searches and citation tracking rather than a complete rerun of a fixed search strategy. Searches were adapted iteratively to the intervention class, crop, molecular endpoint, analytical approach, or safety question being examined. Google Scholar was used primarily for targeted topic searches and citation-oriented discovery within the 2020–2026 date range rather than for exhaustive rank-based screening; no fixed numerical cut-off of Google Scholar results was applied. Duplicate records were identified and removed during reference-library curation in Zotero.

Representative database-specific queries illustrate how the topic-specific searches were implemented. For Scopus, an exemplar query was TITLE-ABS-KEY((“nano-enabled biofortification” OR nanofertilizer* OR “biogenic nanoparticle” OR “biogenic nanoparticles”) AND (“molecular crop quality” OR “nutritional quality” OR metabolom* OR bioaccessib*) AND (crop* OR plant*)), with results limited to articles and reviews published in 2020–2026. For Web of Science Core Collection, an exemplar query was TS = ((“nano-enabled biofortification” OR nanofertilizer* OR “biogenic nanoparticle” OR “biogenic nanoparticles”) AND (“molecular crop quality” OR “nutritional quality” OR metabolom* OR bioaccessib*) AND (crop* OR plant*)), with publication years restricted to 2020–2026 and document types to Article or Review. For PubMed, an exemplar query was ((“nano-enabled biofortification”[Title/Abstract] OR nanofertilizer[Title/Abstract] OR nanofertilizers[Title/Abstract] OR “biogenic nanoparticle”[Title/Abstract] OR “biogenic nanoparticles”[Title/Abstract]) AND (“nutritional quality”[Title/Abstract] OR metabolomic[Title/Abstract] OR metabolomics[Title/Abstract] OR bioaccessibility[Title/Abstract]) AND (crop[Title/Abstract] OR crops[Title/Abstract] OR plant[Title/Abstract] OR plants[Title/Abstract])) AND (“1 January 2020”[Date—Publication]: “31 July 2026”[Date—Publication]). For Google Scholar, targeted combinations such as “nano-enabled biofortification” AND “crop quality” and “biogenic nanoparticles” AND agriculture AND biofortification were used with the 2020–2026 date restriction. These queries are representative examples rather than a single fixed search strategy applied across the entire review.

Priority was given to peer-reviewed primary studies published between 2020 and 2026. Earlier publications were retained when they provided essential conceptual, biochemical, analytical, methodological, or regulatory foundations. Reviews and meta-analyses were used to identify broader trends and additional primary studies, whereas conclusions on intervention efficacy were based preferentially on experimental evidence. Authoritative institutional and regulatory documents were included where relevant. Conference abstracts, theses, and preprints were not retained as core evidence. No formal language-based exclusion criterion was applied; non-English publications were not excluded solely on the basis of language when their scientific content could be adequately assessed.

Studies were prioritized when they linked an agricultural intervention with at least one measurable molecular crop-quality endpoint, including mineral concentration or speciation, gastrointestinal bioaccessibility, primary- or specialized-metabolite profiles, antioxidant-related traits, or transcriptomic, proteomic, ionomic, or metabolomic responses associated with crop composition. Studies reporting only yield, germination, biomass, or general physiological responses were considered only when needed to interpret quality-related effects, physiological injury, or resource efficiency. Following iterative relevance appraisal and reference-library curation, 95 sources were retained and cited in the current manuscript; this literature constituted the evidence base for the critical narrative synthesis rather than a formal systematic-review inclusion set.

Evidence was critically appraised according to intervention identity and formulation, crop and analyzed tissue, experimental system, dose and application route, comparator design, molecular crop-quality endpoint, mechanistic support, formulation characterization, physiological stress, safety and translational evidence, and principal methodological limitations. No numerical quality score, formal risk-of-bias ranking, or predefined exclusion threshold was applied. Evidence was considered more robust when supported by appropriate comparators, dose–response assessment, well-characterized formulations, edible-tissue analysis, complementary molecular or chemical evidence, and reproducibility under biologically or environmentally relevant conditions. Methodologically weaker studies were treated as preliminary rather than automatically excluded. For nano-enabled interventions, interpretation considered material identity and composition, particle size and size distribution, morphology, aggregation state, surface properties, zeta potential where relevant, coating or capping identity, purity, stability, dissolution or transformation behavior, and batch-to-batch reproducibility where available. Incomplete characterization reduced confidence in attributing observed responses specifically to nanoscale properties.

Favorable metabolic regulation was distinguished from nonspecific stress using convergent evidence. Improvements in edible-tissue composition were interpreted more favorably when accompanied by maintained growth and photosynthetic or physiological performance and absence of substantial oxidative injury. Where reported, excessive ROS accumulation, lipid peroxidation, chlorosis, photosynthetic impairment, stress-hormone responses, or stress-responsive transcriptional signatures were considered indicators that increased biochemical activity might reflect physiological stress rather than beneficial metabolic regulation.

## 3. Criteria for Classifying an Agricultural Intervention as Green Chemistry-Compatible

The principles of green chemistry provide a valuable framework for reducing hazardous substances, waste generation, energy demand, and dependence on non-renewable resources [7,8,9]. However, their translation from chemical synthesis to agricultural systems requires caution. Terms such as green-synthesized, biogenic, natural, biodegradable, or nano-enabled describe selected properties of an intervention, but they do not by themselves demonstrate that the intervention is environmentally preferable, safe, or more resource-efficient than a conventional alternative. In agriculture, green chemistry compatibility should therefore be treated as an evidence-based classification rather than as an intrinsic property of a material or technology.

A first criterion concerns the origin and design of the input. Agricultural interventions are more consistent with green chemistry when their production reduces the use of hazardous reagents, relies on renewable or recovered feedstocks, and avoids persistent or toxic stabilizers, coatings, and solvents. Biogenic nanoparticle synthesis using plant extracts, microorganisms, or agricultural by-products may fulfill part of this criterion by replacing conventional reducing and capping agents [12,13,17,33]. Similarly, chitosan-, alginate-, cellulose-, pectin-, or phospholipid-based carriers can reduce dependence on persistent synthetic materials [11]. Nevertheless, biological origin alone does not guarantee safety, because the composition, purity, transformation products, and biological activity of the resulting formulation must still be characterized.

A second criterion is resource efficiency and prevention of nutrient losses. Nano-enabled fertilizers, controlled-release formulations, microbial inoculants, and biostimulants may be considered green chemistry-compatible when they achieve comparable or improved agronomic and quality-related outcomes with lower application rates, reduced leaching or runoff, fewer applications, or improved nutrient use efficiency [34,35,36,37]. Such claims require comparison with an appropriate conventional treatment. An intervention cannot be classified as more resource-efficient merely because it uses a nanoscale or biologically derived formulation if equivalent ionic, chelated, bulk-material, or conventional fertilizer controls are absent.

A third criterion concerns environmental fate, degradability, and safety. The persistence, dissolution, aggregation, transformation, and mobility of an agricultural input determine whether reductions in application rate translate into lower environmental burden. Biodegradability should include not only disappearance of the original carrier but also the identity and safety of its degradation products. For nanoparticle-based systems, characterization of particle size, surface properties, dissolution behavior, soil interactions, plant uptake, and non-target effects is particularly important [16,28,30,38]. An intervention associated with unresolved persistence, ecotoxicity, residue, or trophic-transfer concerns should therefore be described as potentially green chemistry-compatible rather than definitively green.

A fourth criterion is the production of a measurable and nutritionally relevant molecular crop-quality benefit. Improved yield or biomass alone is insufficient within the framework proposed in this review. The intervention should influence endpoints directly relevant to edible-tissue quality, such as micronutrient density, chemical speciation, gastrointestinal bioaccessibility, primary-metabolite balance, specialized-metabolite profiles, or safety-related chemical traits. Increases in phenolics, carotenoids, glucosinolates, capsaicinoids, or antioxidant-related responses may support such classification when they occur without disproportionate growth inhibition, oxidative injury, nutrient imbalance, or deterioration of sensory and postharvest quality [23,39,40,41].

A fifth criterion is comparative and translational robustness. Evidence is stronger when an intervention is evaluated using matched doses, appropriate conventional comparators, well-characterized formulations, edible plant tissues, and field-relevant application routes [27,42,43,44,45]. Reproducibility across genotypes, soils, seasons, or production systems further strengthens the claim [42,43,46,47,48]. Conversely, results obtained from a single crop, short-term controlled conditions, poorly characterized formulations, or unusually high doses should be regarded as proof of concept rather than as evidence of a mature green chemistry solution [42,43,47].

These criteria indicate that green chemistry compatibility is multidimensional. An intervention may perform favorably in one domain but poorly or inconclusively in another. For example, a biogenic nanoparticle may reduce hazardous reagents during synthesis but remain insufficiently characterized in relation to soil persistence; a nanofertilizer may improve nutrient use efficiency but lack evidence of superiority over an ionic formulation; and a biostimulant may increase antioxidant capacity while showing high compositional variability between batches. Green classification should therefore reflect the balance between safer design, resource efficiency, environmental fate, molecular crop-quality, and comparative evidence. These five dimensions, together with the evidence considered sufficient or insufficient to support classification as green chemistry-compatible, are summarized in Table 1. Accordingly, agricultural interventions discussed in this review are considered green chemistry-compatible only when available evidence supports benefits across several of these dimensions without identifying a major unresolved safety or sustainability trade-off. Where evidence is incomplete, the more cautious terms “potentially green chemistry-compatible”, “biogenic”, “biodegradable”, “lower-input”, or “nano-enabled” are used instead of the unqualified term green. This distinction provides the evaluative framework for the following sections on nano-enabled biofortification, biostimulants, biodegradable delivery systems, and molecular crop quality outcomes.

## 4. Evidence for Improving Molecular Crop Quality Through Nano-Enabled Biofortification

Nano-enabled biofortification has been investigated primarily as a means of improving micronutrient delivery to edible plant tissues, but increasing evidence indicates that these interventions may also modify primary and specialized metabolism. Within the framework adopted here, however, improved crop composition alone is insufficient to establish either nutritional superiority or green chemistry compatibility. Evidence must also be considered in relation to formulation characteristics, conventional comparators, dose dependence, physiological condition of the plant, and the nutritional relevance of the measured endpoint [14,15,16]. 

### 4.1. Micronutrient Enrichment and Nutritional Relevance

The most direct evidence for nano-enabled biofortification concerns Zn, Fe, and Se enrichment of edible tissues [16,17,39,40]. ZnO-based formulations have increased Zn accumulation in cereals, vegetables, fruits, and microgreens, although the magnitude of the response varies with crop, application route, dose, and formulation [23,37,50,51,52,53]. Sun et al. [39] provided one of the more informative examples by demonstrating increased Zn concentration in tomato fruit together with changes in amino acid and lipid profiles and improved Zn availability during simulated gastrointestinal digestion. The inclusion of a digestion model strengthens the nutritional interpretation compared with total elemental concentration alone, although simulated assimilation cannot be equated with human absorption or health benefit.

Fe-based nanoformulations have similarly increased Fe accumulation in edible tissues. Phytogenic Fe nanoparticles produced using pomegranate peel extract enhanced Fe accumulation in wheat grain [17], while foliar Fe_3_O_4_ altered Fe distribution within wheat grain [51]. In tomato, direct comparison of nano-Fe with chelated Fe demonstrated formulation-dependent effects on crop performance and quality-related traits [49]. Such comparative designs are particularly important because increases relative to an untreated control cannot distinguish a nanoscale-specific advantage from the effect of supplying the nutrient itself.

Se requires even greater interpretative caution because its nutritional requirement is accompanied by a relatively narrow margin between adequate and excessive exposure. Nano-Se treatment has increased Se accumulation in edible crop tissues, while studies incorporating conventional Se comparators and Se speciation provide a stronger basis for assessing whether nano-enabled delivery offers an actual nutritional advantage [40,54]. Total Se concentration alone is insufficient because nutritional value and safety depend strongly on the chemical forms present.

Across these micronutrients, the principal limitation is therefore not whether nano-enabled treatments can increase elemental accumulation, which they clearly can in selected crop-formulation systems, but whether the additional accumulation represents a meaningful advantage over optimized conventional fertilization. Stronger evidence requires dose-matched comparators together with information on speciation, localization, dissolution behavior, gastrointestinal bioaccessibility, and, ultimately, biological utilization [16,39,54,55].

### 4.2. Effects Beyond Mineral Enrichment: Specialized Metabolites and Functional Quality

Nano-enabled nutrient delivery may also reshape molecular crop quality through changes in specialized metabolites and antioxidant-related traits. These responses are relevant because they indicate that the intervention can influence plant metabolism beyond simple elemental accumulation, but they are highly crop- and dose-dependent [28,50,56].

Li et al. [40], for example, showed that nano-Se application in pepper affected not only Se accumulation but also capsaicin and dihydrocapsaicin, linking micronutrient biofortification with a crop-specific functional quality trait. In rice, foliar SeNP application likewise affected Se availability together with eating-quality traits, extending the assessment of nano-enabled biofortification beyond elemental enrichment alone [57]. Similarly, green-synthesized ZnO nanoparticles modified nutraceutical and antioxidant-related traits in chili pepper fruit in a concentration-dependent manner [58]. Magnesium nanofertilization in green bean increased phenolic and flavonoid contents together with selected bioactive compounds and antioxidant-related responses [41]. Comparative Fe fertilization in tomato likewise produced formulation-dependent changes in lycopene, β-carotene, phenolics, and antioxidant capacity [49]. In broccoli microgreens, combined ZnO and γ-Fe_2_O_3_ nanofertilizers with plant growth-promoting bacterial biofertilizers altered micronutrient accumulation and glucosinolate profiles [23]. Because the latter treatment contained both nano-enabled and microbial components, however, the metabolic response cannot be attributed specifically to the nanoparticles without a factorial separation of the individual interventions.

These examples also illustrate why increases in individual biochemical markers should not automatically be interpreted as improved molecular crop quality. Antioxidant assays such as DPPH, ABTS, and FRAP provide comparative information on reducing or radical-scavenging capacity, but they do not establish bioavailability or health benefit [59]. Similarly, elevated phenolics, proline, antioxidant enzymes, or other stress-responsive compounds may reflect adaptive elicitation or physiological injury. In melon, for example, favorable quality responses to ZnO nanoparticles occurred within a restricted dose range, whereas higher exposure caused chlorosis, impaired photosynthetic performance, and oxidative stress [50]. Molecular crop quality should therefore be considered improved only when desirable compositional changes occur without substantial physiological impairment.

### 4.3. Mechanistic Interpretation: Metabolic Regulation Versus Stress

Mechanistic evidence is necessary to distinguish nutrient-driven metabolic regulation from nonspecific stress responses. Nano-enabled treatments can influence nutrient transport, redox signaling, phytohormone pathways, enzyme activity, and transcriptional regulation, but evidence supporting these mechanisms remains uneven [26,27,28,60,61].

A favorable interpretation is strongest when an improvement in edible-tissue composition is accompanied by maintained growth and photosynthetic performance [49,50], absence of substantial oxidative injury [28,62], and complementary molecular or biochemical evidence [40,63]. Conversely, increases in stress-responsive metabolites or antioxidant activity accompanied by lipid peroxidation, chlorosis, growth inhibition, or other injury markers should not be classified as beneficial metabolic reprogramming [50,60,62]. This distinction is particularly important because moderate ROS generation can participate in signaling and secondary-metabolite induction [28,60], whereas greater exposure may shift the same response toward oxidative damage [50,62].

The integration of metabolomics with ionomics, transcriptomics, or proteomics can strengthen mechanistic interpretation by connecting elemental accumulation with coordinated pathway-level responses [26,61,64]. Recent integrated transcriptomic–metabolomic studies further illustrate the value of linking compositional changes with coordinated molecular responses rather than relying on isolated biochemical endpoints [40,63]. Nevertheless, multi-omics evidence should not itself be treated as proof of causality, because pathway enrichment and cross-omic associations remain inferential unless supported by targeted validation [63,64]. Genotype and developmental stage can modify the biological response to an intervention [65], while sampling time, extraction procedure, analytical platform, normalization, and metabolite-annotation criteria can substantially influence the resulting metabolic interpretation [27,66]. Accordingly, current evidence supports genuine metabolic modulation in selected nano-enabled crop systems but does not justify a general assumption that nanoscale nutrient delivery predictably improves plant metabolism.

## 5. Comparative Appraisal of Intervention Classes

Agricultural interventions proposed as compatible with green chemistry differ substantially in their composition, mechanisms of action, analytical tractability, and maturity of evidence. Nano-enabled delivery systems primarily modify the form, availability, and release of a defined nutrient or active compound. Plant-derived biostimulants and microbial inputs act more indirectly by influencing root development, nutrient acquisition, rhizosphere processes, hormonal regulation, and stress-responsive metabolism. Biodegradable carriers and combined formulations attempt to integrate these functions by improving stability, delivery efficiency, and biological responsiveness. These intervention classes should therefore not be treated as interchangeable or ranked solely according to their effects on yield or individual biochemical markers.

### 5.1. Nano-Enabled Delivery

Nano-enabled delivery is distinguished from the other intervention classes considered here by the relatively high degree of control over nutrient identity, formulation, nominal dose, and physicochemical properties [15,16]. This makes nano-enabled systems particularly suitable for targeted micronutrient delivery and for investigating relationships among formulation characteristics, nutrient uptake, edible-tissue accumulation, and molecular crop quality [14,29]. Among the available approaches, the strongest evidence concerns Zn-, Fe-, and Se-based formulations, for which changes in elemental accumulation and selected biochemical or nutritional endpoints have been demonstrated in edible plant tissues [15,16].

The principal strength of this intervention class is therefore its potential for targeted and formulation-dependent nutrient delivery. In tomato, nanoscale ZnO altered fruit nutrient and metabolite profiles and increased the simulated gastrointestinal availability of selected mineral components [39]. Comparative Fe fertilization further showed that nano- and chelated formulations can produce different effects on phytochemical composition and antioxidant-related traits [49]. Similarly, direct comparison of foliar SeNPs with selenite in rice demonstrated formulation-dependent differences while incorporating Se speciation into the assessment [54]. These findings demonstrate that delivery form can influence molecular crop quality, but they should be interpreted as crop- and formulation-specific rather than as evidence of a general advantage of nanoscale delivery [15,16]. Importantly, direct comparison with a conventional Fe formulation showed that the nanoform was not consistently superior across all measured agronomic and quality endpoints [49].

The main limitation of nano-enabled delivery is the uncertainty surrounding the relationship between the applied material and the biologically active form ultimately encountered by the plant. Nanoparticles may aggregate, dissolve, adsorb to mineral or organic surfaces, or undergo other physicochemical transformations after environmental release [38]. In agricultural systems, such transformations can substantially influence nutrient availability, mobility, plant exposure, and the interpretation of nano-specific effects [29]. Consequently, an observed plant response may arise from intact particles, released ions, transformation products, or a combination of these forms. Without dose-matched ionic, chelated, bulk-material, or conventional controls, it is difficult to distinguish a nanoscale-specific effect from the effect of nutrient supply itself [15,16].

This uncertainty also affects assessment of green chemistry compatibility. Improved uptake efficiency or reduced nutrient inputs may represent a genuine resource-efficiency advantage when demonstrated experimentally [16,29], but such benefits cannot be inferred from nanoscale formulation alone. Environmental persistence, transformation, and potential toxicological effects must be evaluated independently of agronomic efficacy [31,38]. Physicochemical characterization and dissolution behavior are therefore essential for defining the actual exposure generated by the formulation [16], while realistic application rates and comparison with established nutrient sources are required to determine whether the new delivery system provides meaningful practical added value [29].

Overall, nano-enabled delivery currently provides the most direct evidence among the intervention classes considered here for targeted modification of micronutrient-related molecular crop quality [15,16]. At the same time, it requires particularly rigorous material characterization because formulation properties may change substantially after application [38]. Its comparative advantage is therefore most convincing when improved edible-tissue composition or nutrient delivery is demonstrated against an appropriate conventional benchmark [15,49], whereas broader claims of green chemistry compatibility remain premature when environmental transformation, nano-specific effects, and long-term safety remain unresolved [29,31].

### 5.2. Biostimulants and Microbial Inputs

Plant biostimulants comprise substances or microorganisms that stimulate plant nutrition processes independently of their direct nutrient content [19,20]. Non-microbial formulations include plant- and seaweed-derived extracts, protein hydrolysates, and humic substances [67,68,69], whereas microbial inputs include plant growth-promoting bacteria, mycorrhizal fungi, and other beneficial microorganisms associated with the plant–rhizosphere system [21,70]. Compared with nano-enabled nutrient delivery, these interventions are generally less chemically defined and act through broader biological processes, including modification of root development and nutrient acquisition, phytohormone signaling, rhizosphere interactions, osmotic adjustment, and redox regulation [20,21].

Evidence indicates that these processes can extend beyond plant growth to measurable changes in edible-tissue quality. In lettuce, a plant-derived biostimulant improved nutrient-use efficiency together with phenolic composition, ascorbate-related traits, and antioxidant capacity [71]. Recent field evidence in lettuce further showed that non-microbial biostimulants can modify the metabolome together with quality and postharvest traits under agronomically realistic conditions [72]. Chitosan treatment in tomato produced dose-dependent changes in fruit yield and bioactive-compound accumulation, showing that the application level favoring productivity may differ from that maximizing selected molecular crop-quality traits [73]. PGPR-based treatment in soilless tomato similarly modified phenolic accumulation and several physical and nutritional fruit-quality traits [74]. Across horticultural crops more broadly, reported responses include changes in soluble solids, acidity, color, firmness, phenolics, carotenoids, and postharvest quality, although their direction and magnitude remain dependent on crop, formulation, and growing conditions [75]. Recent untargeted metabolomic evidence across cucumber, pepper, and tomato further indicates that biostimulant responses comprise both shared and crop-specific metabolic signatures [76].

Microbial inputs introduce an additional level of context dependence because their effects require successful interaction with the host plant and resident microbiome. Their performance can vary with strain identity, colonization capacity, nutrient status, soil properties, and competition or cooperation within the rhizosphere community [70]. Importantly, evidence from field systems shows that these interactions can influence crop composition as well as plant performance. In a three-season soybean study, bacterial consortia altered rhizosphere microbial structure and functional potential together with plant nutrition, physiology, grain crude protein concentration, and yield [77]. Such results strengthen translational relevance because they demonstrate responses within a functioning soil–plant–microbiome system rather than under a simplified controlled environment. Nevertheless, they also illustrate why causal attribution is more difficult for microbial inputs than for a defined nutrient formulation: changes in crop quality may arise indirectly through nutrient cycling, root development, microbial signaling, or shifts in community function rather than through a single identifiable mechanism.

The principal comparative limitation of this intervention class is therefore reproducibility rather than a lack of biological activity. Protein hydrolysates can vary in feedstock, hydrolysis procedure, peptide-size distribution, free amino acid content, and other bioactive fractions [67], while plant-derived preparations may vary with source material and processing [69,78]. Microbial formulations additionally require strain-level identification, viable-cell quantification, formulation stability, and appropriate inoculum density [44]. Their performance may also depend strongly on compatibility among the inoculant, crop genotype, and soil environment [48]. Consequently, biological or natural origin cannot by itself establish consistent efficacy, reduced environmental burden, or green chemistry compatibility.

Experimental design should reflect this complexity. Because biostimulants are defined by stimulation of plant nutrition processes independently of direct nutrient supply [19,20], a dose-matched mineral-fertilizer comparator is not always the most informative benchmark. Instead, background nutrition should be controlled, and experiments should distinguish the effects of the biostimulant or microbial inoculum from those of carriers, co-formulants, or altered fertilization regimes. Dose–response evaluation remains important [73], while reproducibility across environments and standardized field testing are particularly valuable for microbial products [45]. Where an established agronomic or commercial alternative exists, its inclusion can further clarify whether the intervention provides practical added value.

Overall, biostimulants and microbial inputs offer a broader capacity than nano-enabled delivery to regulate plant and rhizosphere processes simultaneously, and this can translate into improvements in nutrient-use efficiency and selected molecular crop-quality traits [71,74,77]. Their principal strength is therefore biological breadth, whereas their principal weakness is context-dependent reproducibility and limited mechanistic resolution. Within a green chemistry framework, their strongest case arises when a compositionally or biologically well-defined formulation produces reproducible improvements in edible-tissue quality or nutrient-use efficiency under field-relevant conditions, without relying on biological origin alone as evidence of sustainability.

### 5.3. Biodegradable Carriers and Combined Formulations

Biodegradable carriers represent a distinct intervention class because their primary function is not necessarily to supply nutrients or directly stimulate plant metabolism, but to modify the stability, retention, release, and delivery of an active compound. Frequently proposed materials include chitosan, alginate, cellulose-derived polymers, other polysaccharides, lignin-based systems, and phospholipid vesicles [10,11]. From a green chemistry perspective, their potential advantage lies in reducing dependence on persistent synthetic materials and improving delivery efficiency. However, biodegradability alone does not establish environmental superiority: feedstock origin, manufacturing requirements, carrier loading, release behavior, degradation products, and the total material input must also be considered [10].

Evidence connecting biodegradable delivery systems directly with molecular crop quality in edible tissues remains less mature than that available for nano-enabled micronutrient delivery or selected biostimulants. Biodegradable lignin nanoparticles carrying indole-3-acetic acid, for example, were evaluated across tomato development and demonstrated formulation-dependent biological effects, but comprehensive compositional analysis of mature fruit was not performed and higher concentrations produced adverse responses [79]. This illustrates an important distinction between successful delivery and demonstrated food-quality benefit. Similarly, liposomal Se formulations increased Se uptake and enabled assessment of Se speciation in wheat without detectable impairment of major physiological traits [80]. Nevertheless, the study was conducted at the vegetative stage, so enrichment and nutritional relevance of edible grain remain unresolved. Such systems therefore provide convincing proof of delivery functionality, but not yet equivalent evidence of improved molecular crop quality.

A carrier should also be evaluated independently of the active compound it transports. Improved nutrient uptake after encapsulation does not demonstrate that the biodegradable matrix itself confers a sustainability or biological advantage. Robust attribution requires comparison among the free active compound, the loaded formulation and, where biologically relevant, the unloaded carrier. This is particularly important for materials such as chitosan, which may themselves influence plant physiology and specialized metabolism [73]. The comparative value of a carrier therefore lies not in its label as biodegradable, but in evidence that it improves stability, retention, release, or biological availability relative to a suitable non-carrier treatment while avoiding unacceptable additional material or safety burdens [10,11]. Combined formulations extend this concept by coupling complementary modes of action. Nano-enabled components may modify nutrient availability or delivery, whereas microbial or biostimulant components may influence nutrient acquisition, rhizosphere function, root development, or stress regulation [22,81]. Such combinations are attractive because they may integrate the formulation specificity of engineered delivery with the broader biological responsiveness characteristic of microbial or plant-derived inputs. However, greater complexity also makes causal attribution more difficult, and combined formulations should not be assumed to outperform their individual components. In wheat, nanoparticle-coated plant growth-promoting bacteria produced formulation-specific responses, while several combinations did not provide clear improvements over nanoparticles applied alone [82].

**Figure 2:**
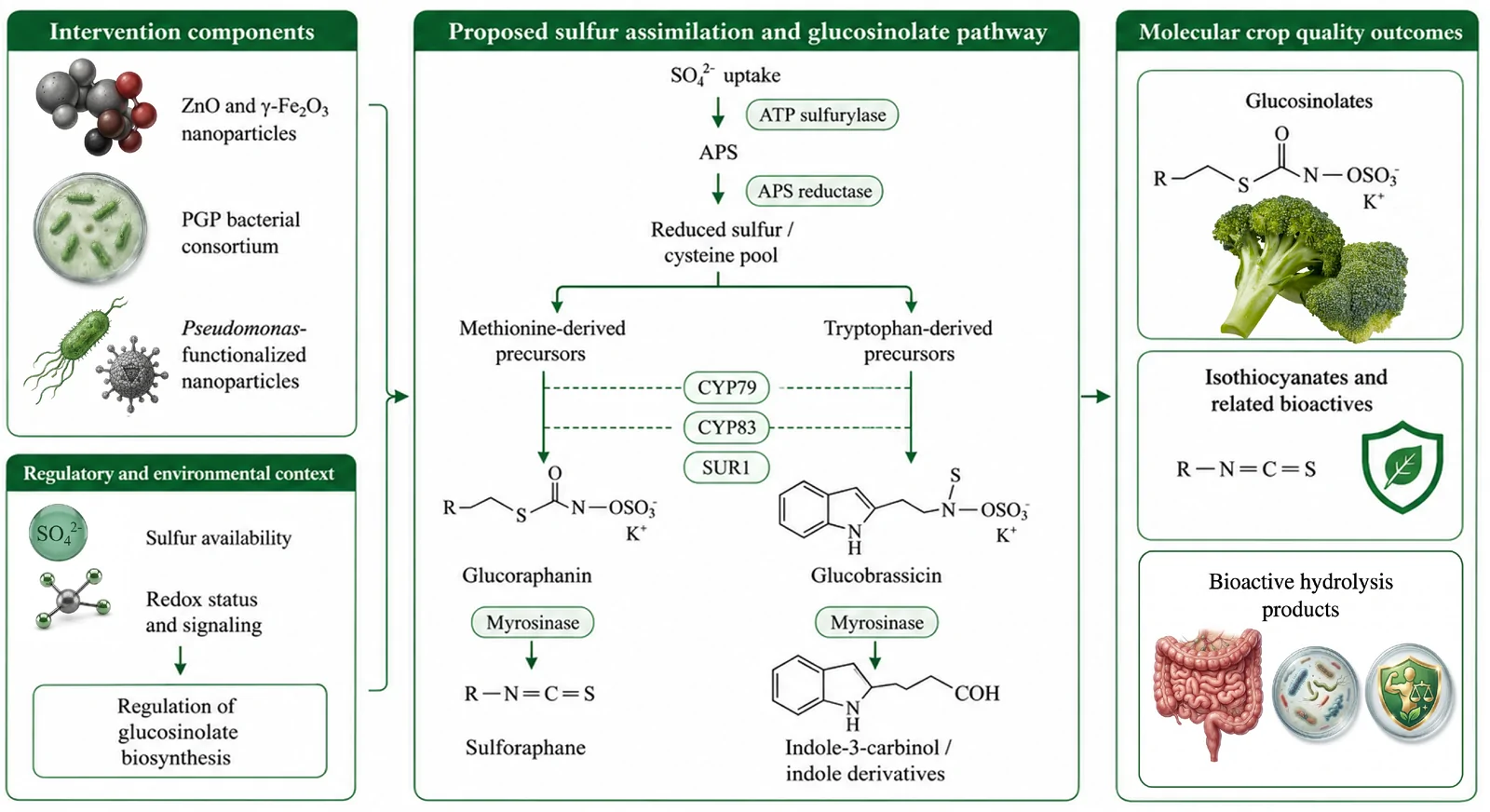
Conceptual interpretation linking combined bio- and nanofertilization with sulfur assimilation and glucosinolate-related molecular crop quality in broccoli microgreens.

Guardiola-Márquez et al. [23] provide a representative example directly relevant to molecular crop quality. Combined ZnO and γ-Fe_2_O_3_ nanofertilizers and plant growth-promoting bacterial treatments in broccoli microgreens increased micronutrient accumulation and modified glucosinolate profiles. These findings demonstrate that nutrient-delivery and microbial processes can converge on edible-tissue composition and provide the experimental basis for the conceptual interpretation shown in Figure 2.

However, because the study did not use a fully crossed factorial interaction model, the observed enhancement should be interpreted as a combined or potentially complementary response rather than definitive synergy [23]. Demonstration of synergy requires explicit comparison of individual components and their combination against a defined additive reference and statistical assessment of interaction [83].

Overall, biodegradable carriers and combined formulations occupy an intermediate position between chemically defined delivery systems and biologically complex biostimulant approaches. Their principal strength is the possibility of integrating controlled delivery with biological responsiveness, whereas their principal limitation is the increasing difficulty of separating carrier, active-compound, and biological-component effects. Within the green chemistry framework proposed here, the strongest evidence would therefore require not only biodegradable or renewable materials, but also a demonstrated delivery advantage, appropriate component-level controls, molecular crop-quality endpoints in edible tissues, and evidence that the additional formulation complexity provides meaningful benefit without introducing disproportionate environmental or safety trade-offs.

### 5.4. Cross-Class Appraisal and Risk–Benefit Trade-Offs

The intervention classes considered in this review differ not only in their mechanisms of action but also in the maturity and type of evidence supporting their compatibility with green chemistry. Nano-enabled delivery currently provides the most direct evidence for targeted modification of micronutrient-related molecular crop quality, particularly for Zn, Fe, and Se [15,16]. Its principal strength is formulation specificity and the possibility of linking material properties with nutrient delivery and edible-tissue composition. This same material specificity, however, creates a comparatively high requirement for physicochemical characterization because aggregation, dissolution, transformation, and interactions within soil–plant systems can alter the identity of the exposure after application [29,38]. Consequently, improvements in crop composition must be weighed against uncertainties concerning nano-specific effects, environmental persistence, and food-chain exposure [31,32].

Environmental assessment should also extend beyond the target crop. Soil microorganisms, mycorrhizal fungi, invertebrates, aquatic organisms receiving agricultural runoff, and other non-target biota may be exposed to agricultural formulations or their transformation products [31,84,85]. Moreover, the absence of detectable effects during a single growing cycle does not exclude accumulation or altered exposure after repeated application. Long-term evaluation should therefore consider residues, transformation products, non-target biological functions, and repeated seasonal use under agronomically realistic conditions [16,29].

Biostimulants and microbial inputs occupy a different position. Their main advantage is biological breadth: they can influence nutrient-use efficiency, root development, stress responses, rhizosphere processes, and specialized metabolism without functioning primarily as direct nutrient sources [20,21,70]. Their principal limitation is not necessarily material persistence but variability and context dependence. Complex composition, batch differences, microbial viability, host compatibility, and interactions with the resident microbiome can reduce reproducibility and make causal attribution more difficult [44,48]. Accordingly, biological origin should not be treated as evidence of intrinsic safety or sustainability. Translation requires well-defined formulations, appropriate quality control, and confirmation that the observed crop-quality response can be reproduced beyond a single controlled environment [45].

Biodegradable carriers and combined formulations potentially bridge these two intervention strategies by coupling controlled delivery with biological responsiveness. Their conceptual advantage lies in improving retention, stability, release, or delivery while reducing reliance on persistent carrier materials [10,11]. However, the evidence base remains less mature at the level of edible-tissue composition, degradation products, and field-scale reproducibility. Se-loaded liposomes illustrate the feasibility of carrier-mediated micronutrient delivery, but the available evidence remains largely proof-of-concept rather than demonstration of improved nutritional quality in harvested grain [80]. Combined nano–microbial systems add further complexity because an enhanced response may arise from additive or complementary effects without constituting true synergy [23,82,83].

These differences mean that no intervention class can be regarded as intrinsically superior or universally compatible with green chemistry. A nano-enabled formulation may be advantageous where a defined micronutrient target requires controlled delivery [15,16], whereas a plant-derived or microbial biostimulant may be more appropriate when the objective is broader improvement of nutrient-use efficiency or physiological resilience [20,21]. A biodegradable carrier may be justified when rapid loss or instability limits the effectiveness of an otherwise useful active compound [10,11]. The relevant comparison is therefore not between technological labels, but between the demonstrated molecular crop-quality benefit and the material, environmental, and safety burden required to achieve it.

This risk–benefit perspective is particularly important for edible crops. Increased elemental concentration does not by itself establish nutritional benefit or food safety because total concentration cannot distinguish among dissolved ions, organic complexes, transformation products, or residual particulate forms [15,32]. Stronger evidence requires integration of total elemental analysis with speciation, transformation or dissolution behavior, tissue localization, and, where relevant, gastrointestinal bioaccessibility [16,86]. The same principle applies beyond nanomaterials. Plant-derived biostimulants require compositional traceability, microbial products require verified identity and viable-cell characteristics [19,44], and biodegradable carriers should be assessed not only for disappearance of the original matrix but also for the identity and safety of degradation products [10,11].

Comparative benchmarking is central to translation because practical added value cannot be established relative to an untreated control alone. The appropriate reference depends on intervention class and should reflect current agronomic practice or an established alternative addressing the same objective. Where the goal is nutritional biofortification, relevant benchmarks may additionally include established genetic, dietary, or food-based strategies targeting the same nutritional deficit [4,87,88].

Taken together, credible translation across intervention classes requires a common evidence framework: a clearly characterized input, an appropriate comparative benchmark, dose–response assessment, molecular crop-quality measurements in edible tissues, evaluation of plant physiological condition, and field-relevant validation. Environmental fate and food-chain safety should be demonstrated independently rather than inferred from descriptors such as nano-enabled, natural, biogenic, or biodegradable. Table 2 summarizes these distinctions by comparing representative studies according to molecular crop-quality endpoints, comparator design, mechanistic support, safety or translational evidence, and the principal limitation affecting interpretation. The studies included in Table 2 were deliberately selected as illustrative cases representing contrasting intervention classes and evidentiary strengths and should not be interpreted as a systematic or exhaustive sample of the literature. Within this framework, the strongest case for green chemistry compatibility arises when an intervention produces a reproducible molecular crop-quality benefit while also reducing input demand, hazardous-material use, or other environmental burdens without introducing disproportionate safety trade-offs.

### 5.5. Translational Implementation: Regulation, Genotype Targeting, and Market Relevance

Regulatory translation requires molecular crop quality to move from a research endpoint toward a defined and verifiable component of product assessment. Current regulatory approaches for nanotechnology-derived materials emphasize formulation identity, physicochemical characterization, exposure, biological behavior, and intended use rather than treating a technological label as evidence of either safety or benefit [30,32]. Comparable traceability is required for microbial and biostimulant products [19,44]. Within the framework proposed here, registration of interventions intended to improve crop quality could therefore be strengthened by incorporating analytically verified edible-tissue endpoints, such as micronutrient concentration and speciation, relevant metabolite profiles, bioaccessibility where appropriate, and evidence that these benefits occur without unacceptable residues, physiological injury, or environmental trade-offs. Such metrics should complement rather than replace conventional efficacy and safety requirements.

Translation also requires recognition that responsiveness is partly a genotype-specific trait. Genotypes differ in nutrient uptake, transport and sequestration, specialized-metabolite biosynthesis, root architecture, and interaction with the rhizosphere microbiome [48,65]. Breeding programs could therefore evaluate not only constitutive nutritional composition but also the stability and magnitude of quality responses to defined nano-enabled, microbial, or biostimulant interventions. Selection should be conducted across representative soils, environments, and developmental stages because intervention performance depends on genotype × environment interactions and agronomic context [43,45,89,90]. Agronomic optimization should likewise consider dose, timing, application route, background nutrient supply, and compatibility with resident microbial communities so that increased molecular crop quality is not achieved at the expense of yield stability, resource efficiency, or safety.

Market translation introduces a further requirement: measurable molecular improvement must also be understandable and valuable to consumers. Studies of biofortified foods indicate that willingness to pay can increase when consumers perceive a credible nutritional benefit, but acceptance depends strongly on prior knowledge, communication, sensory expectations, and the terminology used to describe the product [91,92]. Clear nutrient- or composition-based claims may therefore be more informative than broad descriptors such as green, nano-enabled, or even biofortified, which can be misunderstood or interpreted as production-method claims. Evidence from food sustainability labeling likewise shows substantial heterogeneity in willingness to pay depending on the information communicated [93]. Labeling or certification schemes for molecular crop quality would consequently require standardized analytical thresholds, traceability, reproducible compositional benefits, and independent verification. At present, these approaches should be regarded as a translational opportunity rather than an established certification pathway.

## 6. Cross-Cutting Evidence Limitations and Priorities for Validation

Despite increasing evidence that nano-enabled fertilizers, biostimulants, microbial inputs, and biodegradable delivery systems can modify molecular crop quality, direct comparison among studies remains difficult. The principal sources of uncertainty are not restricted to any single intervention class but arise from differences in exposure definition, comparator design, biological context, analytical workflow, and the strength of mechanistic inference [16,29,42]. These limitations determine whether a reported compositional change can be attributed confidently to the intervention and whether it is likely to remain reproducible under agronomically relevant conditions.

### 6.1. Exposure Definition, Comparator Design, and Formulation Reproducibility

A meaningful comparison requires that the applied intervention be sufficiently characterized to define the biological exposure. For nano-enabled systems, nominal elemental composition is insufficient because particle size distribution, surface properties, aggregation, coating, stability, and dissolution can affect nutrient availability and biological activity [16,29]. Similar problems occur with biologically derived formulations. Protein hydrolysates may differ substantially in raw material, hydrolysis conditions, peptide distribution, and free amino acid content [67], whereas microbial products require strain-level identification, viable-cell quantification, inoculum density, and formulation stability [44].

Dose is equally important because biological responses are frequently non-linear. An application level that stimulates nutrient acquisition or secondary metabolism may not coincide with the dose maximizing yield, and excessive exposure can shift the response toward physiological stress [28,73]. Evidence based on a single concentration should therefore be interpreted primarily as proof of concept rather than as definition of an effective application range.

Comparator design determines the strength of the resulting claim. An untreated control demonstrates treatment responsiveness but does not establish superiority over existing practice. Nano-enabled nutrient formulations should therefore be compared, where possible, with dose- and nutrient-matched ionic, chelated, bulk, or conventional sources [16,29]. Carrier systems additionally require separation of the free active compound from the loaded formulation and, where the carrier is biologically active, from the carrier itself [10,80]. For combined formulations, claims of synergy require individual components, their combination, and an explicit reference model against which departure from additivity can be tested statistically [83]. Otherwise, responses should be described more cautiously as additive, complementary, or formulation-specific.

Across intervention classes, robust attribution therefore depends on a clearly defined exposure, an appropriate comparator, and sufficient formulation reproducibility. Without these elements, differences among experiments may reflect variation in the tested material rather than genuine differences in crop response.

### 6.2. Biological Context and Field Reproducibility

Intervention effects are strongly conditioned by crop genotype, developmental stage, tissue identity, application route, and production environment. Genotype can influence nutrient transport, sequestration, antioxidant regulation, root architecture, and specialized-metabolite biosynthesis, while environmental conditions modify many of the same processes through gene-by-environment interactions [65]. Consequently, responses observed in young vegetative tissues, microgreens, or hydroponic systems cannot be assumed to predict nutrient redistribution and metabolite accumulation in mature edible organs.

Application route further changes exposure. Foliar delivery is influenced by leaf age, cuticular properties, spray retention, humidity, and the interval between treatment and harvest, whereas soil-applied inputs interact with pH, organic matter, mineral surfaces, moisture, and microbial activity [16]. These dependencies are especially important for microbial formulations because colonization and persistence are determined jointly by the inoculum, host genotype, soil properties, and resident microbial community [45,48].

Controlled experiments remain valuable for identifying mechanisms, but translational confidence increases substantially when responses are reproduced under realistic field conditions. Current guidelines for enhanced-efficiency fertilizers emphasize agronomically representative management and appropriate field comparisons [43], while microbial-input research increasingly calls for standardized field testing rather than extrapolation from single controlled environments [45]. Open-field studies also demonstrate that introduced microbial consortia interact with native rhizosphere communities rather than functioning independently of them [89]. Meta-analytical evidence further indicates substantial heterogeneity in biofertilizer responses across crops and field environments [90].

The relevant unit of interpretation is therefore not the intervention alone but the intervention × genotype × environment × developmental stage combination. Evidence should be considered strongest when molecular crop-quality responses are reproduced across cultivars, environments, or seasons rather than inferred from one experimental context.

### 6.3. Analytical Reliability and Strength of Mechanistic Evidence

Analytical heterogeneity represents a separate source of uncertainty. Molecular crop-quality measurements depend on sampling strategy, tissue stabilization, storage, extraction, analytical platform, calibration, normalization, and metabolite-identification criteria [27,66]. Differences introduced before instrumental analysis may be as important as the choice of analytical platform itself. Reporting on a fresh- versus dry-weight basis also requires care when treatments alter tissue hydration or biomass accumulation.

Extraction methods should therefore be evaluated according to both analytical performance and sustainability. Reduced solvent use or shorter processing time is advantageous only when recovery, selectivity, compound stability, and reproducibility remain adequate [94]. Likewise, analytical platforms provide complementary rather than interchangeable information. Total elemental analysis can quantify enrichment but cannot establish chemical form, localization, or particulate state [16]. Particle-resolved approaches such as single-particle ICP-MS may add information on particulate fractions, although extraction efficiency, matrix interference, and material transformation during sample preparation can complicate interpretation [95]. Spectrophotometric antioxidant assays remain useful for comparative screening, but differences among DPPH, ABTS, FRAP, and related methods mean that increased assay values should not be interpreted directly as enhanced nutritional or physiological benefit [59].

Mechanistic claims require similar caution. Coordinated changes in transcripts, metabolites, proteins, or elemental profiles can substantially strengthen interpretation, particularly when multiple analytical layers converge on the same pathway [61,64]. Integrated transcriptomic–metabolomic studies illustrate the potential of this approach for linking nutrient treatment with pathway-level responses in edible tissues [63]. Nevertheless, cross-omic correlation and pathway enrichment remain inferential unless supported by targeted validation, temporal resolution, or functional evidence.

Accordingly, terms such as pathway activation, metabolic reprogramming, or mechanistic regulation should be reserved for evidence that goes beyond compositional association. In many studies, the more defensible conclusion is that an intervention is associated with coordinated molecular and biochemical responses, rather than that a specific causal pathway has been demonstrated.

Overall, these limitations define a practical hierarchy of evidence. Confidence is lowest when conclusions rely on a single dose, poorly characterized formulation, unmatched control, one experimental environment, or isolated biochemical endpoint. It increases when exposure is well defined, appropriate comparators and dose ranges are included, edible tissues are analyzed with validated methods, physiological injury is excluded, and molecular findings are reproduced across relevant biological or environmental contexts. This hierarchy should guide interpretation of current studies and the design of future work aimed at establishing reproducible molecular crop-quality benefits rather than treatment-specific proof of concept.

## 7. Conclusions

Molecular crop quality should be recognized as a necessary endpoint of sustainable agriculture rather than as a secondary consequence of crop production. Yield, input reduction, and environmental performance remain essential, but they do not establish whether an agricultural system produces food with improved nutritional composition, bioaccessibility, functional value, and chemical safety. The framework proposed in this review therefore extends green chemistry assessment from the design of agricultural inputs to the measurable composition of edible plant tissues.

The strongest current evidence concerns nano-enabled delivery of Zn, Fe, Se, and selected other nutrients in defined crop-formulation systems. These interventions can increase micronutrient density and, in some cases, modify metabolite profiles, antioxidant-related traits, and simulated gastrointestinal bioaccessibility. Biostimulants and microbial inputs also show potential to improve nutrient-use efficiency and selected molecular crop-quality traits, but their effects are more strongly influenced by formulation composition, crop genotype, environmental conditions, and batch variability. Biodegradable carriers and combined bio–nano systems remain promising, although evidence for their field reproducibility, degradation behavior, and comparative superiority is still limited.

No intervention class can therefore be considered intrinsically green. Green chemistry compatibility requires evidence of safer input design, improved resource efficiency, a nutritionally relevant molecular crop-quality benefit, acceptable environmental and food-safety profiles, and comparative performance against an appropriate conventional alternative. At present, most technologies satisfy only part of this framework and should be described as potentially or partially green chemistry-compatible.

The main barriers are inadequate dose-matched comparators, incomplete formulation characterization, analytical heterogeneity, uncertain attribution of effects in combined treatments, and the predominance of short-term controlled-environment studies. Future research should determine whether compositional improvements are reproducible across genotypes, soils, seasons, and realistic field conditions and whether they remain beneficial when bioaccessibility, physiological stress, environmental fate, and food-chain exposure are considered together. Molecular crop quality thus provides a critical bridge between green chemistry, plant metabolism, food chemistry, and sustainable agriculture, allowing innovations to be judged not only by what they reduce or avoid, but also by the nutritional and chemical quality they reliably and safely produce.

## Figures and Tables

**Table 1:** Evidence-based criteria proposed for classifying agricultural interventions as green chemistry-compatible. The criteria were synthesized from green chemistry principles and current evidence on agricultural nanotechnology, biostimulants, molecular crop quality, and environmental safety.

Criterion	Evidence Supporting Green Chemistry Compatibility	Insufficient Basis for Classification	Representative References
**Safer input** ** design**	Reduced hazardous reagents, renewable feedstocks, characterized composition	Biological or natural origin alone	[7,8,10,11,12,13,17,33]
**Resource** ** efficiency**	Lower dose, reduced losses, fewer applications, appropriate conventional comparator	Improved uptake without dose-matched comparison	[34,35,36,37,39,49]
**Environmental** ** fate and safety**	Degradability, transformation, residue and non-target assessment	Assumed safety based on biogenic synthesis	[16,28,31,38]
**Molecular ** ** crop quality**	Improvement in edible-tissue composition, bioaccessibility or relevant metabolite profiles	Yield or biomass increase alone	[23,39,40,41]
**Translational** ** robustness**	Field-relevant dose, reproducibility, matched controls and formulation characterization	Single short-term controlled experiment	[16,29,39,49]

**Table 2:** Critical appraisal of representative primary studies evaluating agricultural interventions in relation to molecular crop quality and green chemistry compatibility.

Intervention	Crop/ Model	Molecular Quality Endpoint	Comparator Design	Mechanistic Support	Safety/Translation Evidence	Critical Limitation
**Nanoscale** **ZnO [39]**	Tomato fruit	Fruit Zn; amino acid and lipid profiles; composition of intestinal-fluid fractions after simulated digestion	Control treatment; no clearly resolved dose-matched conventional Zn benchmark	Elemental analysis and metabolomic profiling; no direct causal pathway validation	Edible fruit and simulated gastrointestinal digestion	The in vitro model does not establish human absorption; the nano-specific advantage and field reproducibility remain uncertain.
**Nano-Fe and chelated Fe [49]**	Tomato fruit under drip-irrigated plasticulture	Vitamin C, lycopene, carotenoids, flavonoids, polyphenols, antioxidant capacity, and marketable yield	Direct factorial comparison of nano-Fe and chelated Fe at 0, 10, 20, and 40 mg L^−1^	Compositional and physiological measurements supported by multivariate analysis; no direct molecular mechanism	Multiple doses and marketable fruit assessed in a production-relevant system	Nano-Fe was not consistently superior; chelated Fe at 20 mg L^−1^ produced the strongest response for several phytochemical endpoints.
**ZnO and γ-Fe_2_O_3_** ** nanofertilizers with PGP** ** bacterial** ** treatments [23]**	Broccoli microgreens	Zn and Fe accumulation, glucosinolate profiles, and growth traits	Multiple nanoparticle, mineral-precursor, bacterial, functionalized, and combined treatments; no fully crossed factorial interaction model	Compositional evidence; the pathway-level interpretation remains conceptual	Edible microgreens assessed under controlled seedbed conditions	One genotype, three seedbed replicates, and a 12-day growth period; statistical interaction and universal synergy were not demonstrated.
**Chitosan [73]**	Tomato fruit	Yield, vitamin C, phenolic compounds, carotenoids, and color-related traits	Untreated control and several application doses	Elicitation and dilution effects were proposed, but the underlying pathway was not directly validated	Dose–response relationship and edible fruit quality assessed	The dose favoring yield differed from that favoring selected molecular crop-quality traits, and responses were influenced by changing environmental conditions.
**Se-loaded** **liposomes [80]**	Wheat plants at the vegetative stage	Se uptake and speciation, biomass, pigments, and mineral composition	Liposomal Se formulations compared with direct Se application, using different phospholipids, Se forms, and concentrations	X-ray absorption spectroscopy supported Se speciation and biotransformation; no plant molecular mechanism was tested	Se enrichment occurred without detectable impairment of biomass, pigments, or mineral-nutrient status	Proof of concept was limited to vegetative plants; Se enrichment of grain, gastrointestinal bioaccessibility, and field performance were not assessed.
**Green leaves** **biostimulant [71]**	Lettuce leaves	Phenolics, ascorbate, antioxidant capacity, nitrate, soluble sugars and proteins, nutrient uptake, and nutrient-use efficiency	Untreated control and two doses, 3 and 5 mL L^−1^	Photosynthetic performance, nutritional status, and nutrient efficiency were assessed, but effects of individual formulation components were not resolved	Edible leaves, two application doses, photosynthetic performance, and nitrate accumulation assessed	A single complex formulation was evaluated under optimal growth conditions; component attribution and field reproducibility remain unresolved.

## Data Availability

No new data were created or analyzed in this study. Data sharing is not applicable to this article, as this is a review based on the previously published literature.
